# Heterogeneous behavior of lipids according to HbA1_c_ levels undermines the plausibility of metabolic syndrome in type 1 diabetes: data from a nationwide multicenter survey

**DOI:** 10.1186/1475-2840-11-156

**Published:** 2012-12-27

**Authors:** Fernando MA Giuffrida, Alexis D Guedes, Eloa R Rocco, Denise B Mory, Patricia Dualib, Odelisa S Matos, Reine M Chaves-Fonseca, Roberta A Cobas, Carlos Antonio Negrato, Marilia B Gomes, Sergio A Dib

**Affiliations:** 1 CEDEBA-Centro de Endocrinologia do Estado da Bahia, Salvador, Brazil; 2 Diabetes Center, Federal University of Sao Paulo, Sao Paulo, Brazil; 3 Rio de Janeiro State University, Rio de Janeiro, Brazil; 4 Associação de Diabéticos de Bauru, Bauru, Brazil

**Keywords:** Type 1 diabetes, Metabolic syndrome, Dyslipidemia, Cardiovascular risk factor

## Abstract

**Background:**

Cardiovascular risk factors (CVRF) may cluster in type 1 diabetes, analogously to the metabolic syndrome described in type 2 diabetes. The threshold of HbA1_c_ above which lipid variables start changing behavior is unclear. This study aims to 1) assess the behavior of dyslipidemia according to HbA1_c_ values; 2) detect a threshold of HbA1_c_ beyond which lipids start to change and 3) compare the clustering of lipids and other non-lipid CVRF among strata of HbA1_c_ individuals with type 1 diabetes.

**Methods:**

Effects of HbA1_c_ quintiles (1st: ≤7.4%; 2nd: 7.5-8.5%; 3rd: 8.6-9.6%; 4th: 9.7-11.3%; and 5th: >11.5%) and covariates (gender, BMI, blood pressure, insulin daily dose, lipids, statin use, diabetes duration) on dyslipidemia were studied in 1275 individuals from the Brazilian multi-centre type 1 diabetes study and 171 normal controls.

**Results:**

Body size and blood pressure were not correlated to lipids and glycemic control. OR (99% CI) for high-LDL were 2.07 (1.21-3.54) and 2.51 (1.46-4.31), in the 4th and 5th HbA1_c_ quintiles, respectively. Hypertriglyceridemia increased in the 5th quintile of HbA1_c_, OR 2.76 (1.20-6.37). OR of low-HDL-cholesterol were 0.48 (0.24-0.98) and 0.41 (0.19-0.85) in the 3rd and 4th HbA1_c_ quintiles, respectively. HDL-cholesterol correlated positively (0.437) with HbA1_c_ in the 3rd quintile. HDL-cholesterol and insulin dose correlated inversely in all levels of glycemic control.

**Conclusions:**

Correlation of serum lipids with HbA1_c_ is heterogeneous across the spectrum of glycemic control in type 1 diabetes individuals. LDL-cholesterol and triglycerides worsened alongside HbA1_c_ with distinct thresholds. Association of lower HDL-cholesterol with higher daily insulin dose is consistent and it points out to a role of exogenous hyperinsulinemia in the pathophysiology of the CVRF clustering. These data suggest diverse pathophysiological processes depending on HbA1_c_, refuting a unified explanation for cardiovascular risk in type 1 diabetes.

## Background

Fatal cardiovascular disease before 40 years old shows an almost 20-fold increase in patients with type 1 diabetes compared with non-diabetic individuals [1]. Dyslipidemia is a key cardiovascular risk factor (CVRF) in type 1 diabetes, with tighter treatment goals than the non-diabetic population [2]. Nevertheless, differences in the behavior of dyslipidemia in type 1 diabetes are not only quantitative but qualitative when compared with non-diabetic individuals. Although CVRF increase in the general population alongside increasing glucometabolic disturbances [3], some evidence point to lipid profile being globally worsened in type 1 diabetes, with lower HDL-cholesterol and higher LDL and triglycerides [4], whereas others have demonstrated higher HDL-cholesterol levels [5], albeit with a less protective profile [6]. Besides, some studies have shown lipids to take a less important role in the increased CV risk of type 1 diabetes than other factors [7,8]. Furthermore, type 1 diabetes seems to attenuate or even erase gender differences in cardiovascular (CV) disease [5]. Although observational studies have shown improved lipid profile with better glycemic control [9], there is uncertainty about the role of improved glycemic control in the prevention of macrovascular disease in these patients, as there are no well-defined thresholds of HbA1_c_ beyond which lipid levels begin to change in type 1 diabetes [4,10,11].

Clustering of CVRF may occur in type 1 diabetes [12,13], analogously to the one known as metabolic syndrome in type 2 diabetes, but there is a different multifactorial pathophysiology for the clustering in each of the two major types of diabetes [12,14,15]. Currently, a unified explanation for the buildup of CVRF with the progression of type 1 diabetes is debatable as a pathological entity, regarding its prognostic importance. In addition to the intricate interaction among CVRF, hyperinsulinemia caused by exogenous insulin replacement can add to the complexity of this scenario.

Factor analysis (FA) is a statistical method that provides correlation coefficients (called factor loadings) of studied variables with latent variables called factors, rather than among variables themselves. It has been frequently used to gain insight on the clustering of CVRF of type 2 diabetes [16]. This method has been criticized for its low reproducibility, since results depend on which variables are entered in the models [17]. While some FAs have suggested the existence of a single factor responsible for the clustering of CVRF in type 2 diabetes [18,19], others have shown the existence of a lipid factor (correlated to HDL-cholesterol and triglycerides), an insulin resistance (IR) factor (blood glucose and insulin), and a body size factor (BMI and abdominal circumference) in both non-diabetic and type 2 diabetic individuals [20,21]. Others have shown association of lipid variables with glycemic control [22], albeit not employing HbA1_c_. In type 1 diabetes, a previous FA has found roughly the same type 2 diabetes classical factors, but without assessing glycemic control in these models [23]. In another FA of CVRF in individuals with type 1 diabetes, we previously showed HbA1_c_ to have a continuous correlation with lipid variables and to disrupt the classical lipid factor when employed [24], suggesting the grouping of lipids and CVRF to vary across the wide glycemic control range displayed by individuals with type 1 diabetes. Since FA results are heavily influenced by which variables are entered in the models, the question of how glucose metabolism influences clustering of lipids and other CVRF is still open in type 1 diabetes.

We hypothesize that the heterogeneity in the clustering of lipids and other CVRF seems to arise partly from the heterogeneity in the concept of the cluster itself in individuals with diabetes mellitus, as recently discussed by Reaven [14]. Given the different results obtained in FAs of CVRF clusters in different populations [25], the clustering of lipid abnormalities and other non-lipid CVRF may not have the same meaning in different clinical settings.

In this study, we aimed to: 1) assess the behavior of dyslipidemia according to HbA1_c_ values individuals with type 1 diabetes; 2) detect a threshold of HbA1_c_ beyond which lipids start to change; 3) compare the clustering of lipids and other non-lipid CVRF among strata of HbA1_c_.

## Methods

Data from the Brazilian Type 1 Diabetes Study Group (BrazDiab1SG) have been analyzed. In brief, BrazDiab1SG is a multi-centre cross-sectional study of a population-based sample representative of individuals with type 1 diabetes from all 5 major geographical regions of Brazil, totaling 3591 patients. Research methodology has been described in detail elsewhere [26]. Individuals with available data on HbA1_c_ measured by a NGSP standardized method (with normal range 4-6%) and age equal or older than 12 years old have been selected for this study, resulting in a sample of 1275 patients.

The following variables were studied: gender, age, diabetes duration (log-transformed to approximate normal distribution), BMI, mean blood pressure (MBP), calculated as the sum of one-third of systolic blood pressure and two-thirds of diastolic blood pressure, total cholesterol, HDL-cholesterol, LDL-cholesterol, triglycerides, HbA1_c_, total insulin dose, statin use, smoking, microalbuminuria, and presence of nephropathy (defined as any degree of abnormality from macroalbuminuria to decreased renal function, but excluding overt renal failure). Lipids have been dichotomized according to the NCEP-ATP III criteria [27]: low-HDL-cholesterol was defined as HDL-cholesterol ≤ 1.04 mmol/L (≤ 40 mg/dL in conventional units), high-LDL-cholesterol as LDL-cholesterol ≥ 2.59 mmol/L (≥ 100mg/dL), and hypertriglyceridemia as triglycerides ≥ 1.7 mmol/L (≥150 mg/dL). HbA1_c_ values have been divided in quintiles. Continuous variables have been compared using ANOVA with Scheffe’s post-hoc testing. Categorical variables have been compared by Chi-square.

Low-HDL-cholesterol, high-LDL-cholesterol, and hypertriglyceridemia have been entered as dependent variables in forward logistic regression models, with HbA1_c_ quintiles as independent categorical covariate, using the 1st quintile as reference category. All other studied variables have been entered in different combinations in continuous or categorical form, the best fit for each model being chosen according to Nagelkerke pseudo-R-squared values and Hosmer-Lemeshow goodness-of-fit test. Values were recorded as OR (99% CI).

Exploratory factor analyses of age, diabetes duration (log-transformed), total daily insulin dose, MBP, HDL-cholesterol, triglycerides, and HbA1_c_ have been performed separately by quintiles of HbA1_c_. Oblique rotation has been employed in order to achieve simple structure. Factor analysis has also been performed in 171 non-diabetic controls, utilizing the same variables as in type 1 diabetic individuals, except for serum fasting insulin in place of insulin dose to account for the role of hyperinsulinemia/IR and the exclusion of diabetes duration, which has no correlate parameter in non-diabetic individuals. Baseline characteristics of this group have been previously described [28].

Statistical analyses have been carried out with SPSS 13.0 for Windows software (SPSS Inc., Chicago, IL, USA). Graphics have been made in OmniGraphSketcher 1.2.1 (v22.23) for MacOS (The Omni Group, Seattle, WA, USA).

The study was approved by the local Ethics Committee of each participant institution, as previously described [26].

## Results

Baseline characteristics of participants according to HbA1_c_ quintiles are described in Table 1, first only with univariate analysis. 

**Table 1:** **Clinical and laboratory characteristics of individuals with type 1 diabetes, divided by HbA1**_**c**_**quintiles**

	**HbA1**_**c**_** quintiles**					
	**1st**	**2nd**	**3rd**	**4th**	**5th**	
	**≤ 7.4%**	**7.5 - 8.5%**	**8.6 - 9.6%**	**9.7 - 11.4%**	**≥ 11.5%**	**p**
**n**	**255**	**258**	**255**	**255**	**252**	
Age (years)	26.1 ± 12.0	26.7 ± 11.6	25.2 ± 11.4	23.9 ± 10.5	20.9 ± 9.1	< 0.05^a^
Duration of diabetes (years)	12.2 ± 10.1	12.9 ± 8.7	11.9 ± 7.9	10.5 ± 7.5	8.9 ± 6.4	< 0.05^b^
Total daily insulin dose (U)	45.6 ± 19.9	52.7 ± 22.8	55.2 ± 20.0	58.5 ± 21.5	62.1 ± 23.5	< 0.05^c^
BMI (kg/m^2^)	22.5 ± 3.7	23.2 ± 3.9	23.1 ± 3.9	22.8 ± 4.4	21.7 ± 3.8	< 0.05^d^
HbA1_c_ (%)	6.6 ± 0.64	8.0 ± 0.32	9.1 ± 0.33	10.5 ± 0.55	13.6 ± 1.77	< 0.05^e^
Fasting plasma glucose (mmol/L)	7.77 ± 4.12	9.30 ± 5.23	10.68 ± 5.81	11.22 ± 5.81	12.96 ± 7.37	< 0.05^f^
Mean Blood Pressure (mmHg)	101.9 ± 12.8	100.9 ± 12.9	102.1 ± 13.4	101.2 ± 13.2	99.7 ± 12.8	NS
Total cholesterol (mmol/L)	4.08 ± 1.03	4.21 ± 1.05	4.34 ± 1.13	4.57 ± 1.01	4.86 ± 1.19	< 0.05^g^
HDL-cholesterol (mmol/L)	1.34 ± 0.46	1.38 ± 0.38	1.37 ± 0.34	1.43 ± 0.41	1.38 ± 0.37	NS
LDL-cholesterol (mmol/L)	2.31 ± 0.78	2.42 ± 0.79	2.53 ± 0.88	2.67 ± 0.86	2.90 ± 0.99	< 0.05^h^
Triglycerides (mmol/L)	0.92 ± 0.64	0.95 ± 0.72	1.00 ± 0.72	1.04 ± 0.70	1.31 ± 1.18	< 0.05^a^
Male gender (%)	49.0	46.9	42.7	39.6	36.9	0.034^i^
Statin users (%)	10.2	14.7	10.6	7.5	5.6	0.007^i^
Hypertriglyceridemia (%)	7.6	8.5	12.3	13.9	25.2	< 0.001^i^
Low HDL-cholesterol (%)	21.8	14.0	12.6	10.0	16.6	0.008^i^
High LDL-cholesterol (%)	31.6	34.2	40.4	50.0	57.2	< 0.001^i^
Microalbuminuria (%)	44.7	56.6	51.4	47.5	45.6	< 0.044^i^
Smokers (%)	4.7	6.6	4.7	2.4	6.7	NS

^a^ 5th quintile vs. all others; ^b^ 5th vs. 1st, 2nd, 3rd and 2nd vs. 4th quintiles; ^c^ 1st vs. all others, 2nd vs. 4th and 5th, 3rd vs. 5th quintiles; ^d^ 5th vs. 2nd, 3rd and 4th quintiles; ^e^ all pairwise comparisons; ^f^ 5th vs. all others; 1st vs. 3rd, 4th, 5th; 2nd vs. 4th quintile; ^g^ 1st vs. 4th and 5th; 2nd vs. 4th and 5th; 3rd vs. 5th quintiles; ^h^ 5th vs. 1st, 2nd, and 3rd; 4th vs. 1st quintiles; ^i^ 4 degrees-of-freedom significance; NS non-significant.

**Figure 1:**
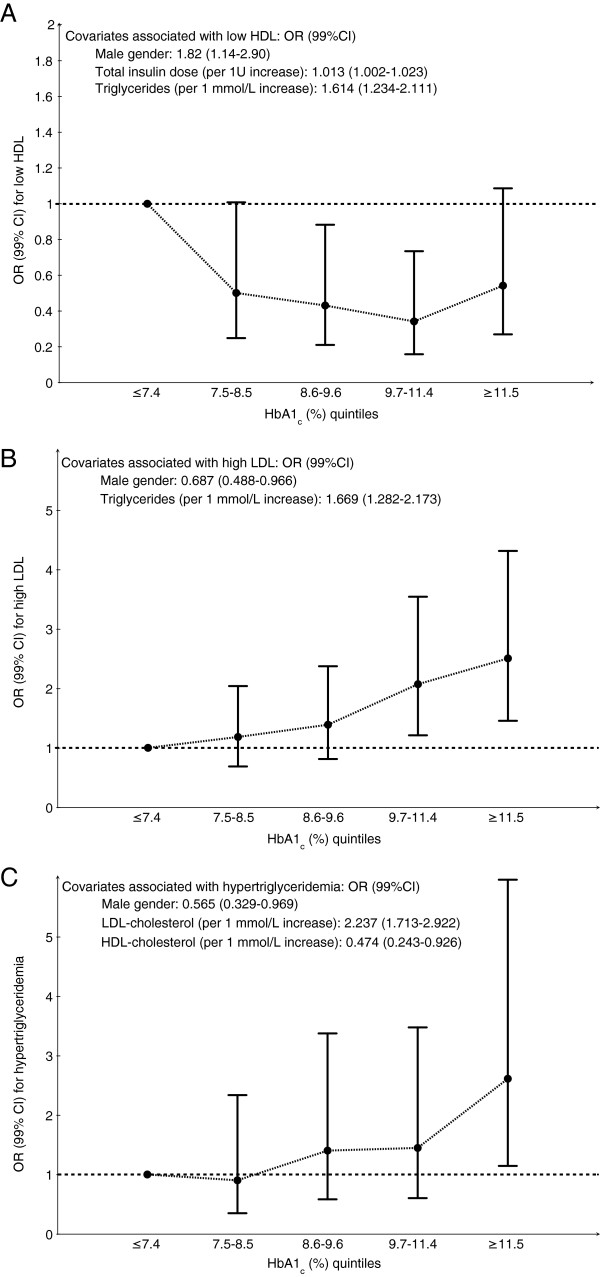
**OR (99% CI) of dyslipidemia according to HbA1**_**c**_**quintile.A**. Low-HDL-cholesterol (HDL-cholesterol ≤ 1.04 mmol/L) **B**. High-LDL (LDL ≥ 2.59 mmol/L). **C**. Hypertriglyceridemia (triglycerides ≥ 1.7 mmol/L). All ORs are relative to the reference category (1st quintile).

Logistic regression models with ORs (99% CI) of dyslipidemia (as categorical variables) per HbA1_c_ quintile are depicted in Figure 1A (low-HDL), B (high-LDL), and C (hypertriglyceridemia). All models were adjusted for age, diabetes duration, total daily insulin dose, statin use, BMI, blood pressure, presence of microalbuminuria and/or overt nephropathy, and smoking. Low-HDL-cholesterol was significantly less frequent in the 3rd HbA1_c_ quintile, with OR 0.48 (0.24-0.98), and in the 4th quintile, with OR 0.41 (0.19-0.85) when compared to the 1st quintile. Covariates associated to low-HDL-cholesterol were male gender (OR 1.82 [1.14-2.90]), total insulin dose (OR 1.013 [1.002-1.023] for each 1U increase), and triglycerides (OR 1.614 [1.234-2.111] for each 1 mmol/L increase). High-LDL-cholesterol had significant higher ORs in the 4th and 5th quintiles, respectively 2.07 (1.21-3.54) and 2.51 (1.46-4.31) when compared to the 1st quintile. Covariates associated with high-LDL were male gender (OR 0.687 [0.488-0.966]) and triglycerides (OR 1.669 [1.282-2.173] per each 1 mmol/L increase). Hypertriglyceridemia increased only in the 5th quintile of HbA1_c_, with OR 2.76 (1.20-6.37). Significant covariates were male gender (ORs 0.565 [0.329-0.969]), LDL-cholesterol (2.237 [1.713-2.922] per 1 mmol/L increase), and HDL-cholesterol (0.474 [0.243-0.926] per 1 mmol/L increase). Other covariates were not associated to dyslipidemia in any of the models. Although the presence of microalbuminuria was different among HbA1_c_ quintiles in univariate analysis, this difference was not maintained in various multivariate models tested. 

**Figure 2:**
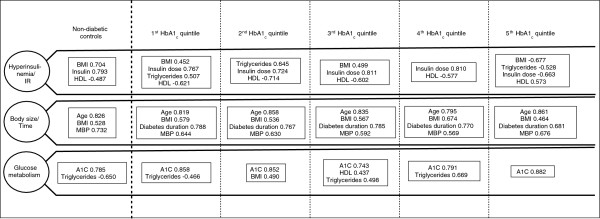
**Comparison of factor structures of CVRF clusters in 171 non-diabetic controls and 1275 individuals with type 1 diabetes divided by quintiles of HbA1**_**c**_**(signs of correlation coefficients are valid only to compare variables within the same factor and in the same group).**

Factor analysis has extracted three factors uniformly in all HbA1_c_ quintiles and in the non-diabetic control group. Factors have been denominated Hyperinsulinemia/IR, Body Size/Time, and Glucose Metabolism. The three factors along with factor loadings for significant variables (those with coefficients above 0.40) are depicted schematically in Figure 2. The Hyperinsulinemia/IR factor has correlated directly with insulin daily dose and inversely with HDL-cholesterol constantly in all five groups of individuals with type 1 diabetes. A similar correlation occurred in normal controls with fasting serum insulin. The Body Size/Time factor has showed a constant structure of BMI, age, MBP, and diabetes duration (except in non-diabetic individuals, in whom this variable is not applicable) in all six groups. Glucose Metabolism factor showed constant correlation to HbA1_c_ in all groups, with loadings in the 0.7-0.9 range in all groups, but correlation of lipids with this factor was variable. Of note, in non-diabetic controls and in the 1st HbA1_c_ quintile, correlations of HbA1_c_ and triglycerides occurred in opposite directions (i.e., decreasing triglyceride levels with increasing HbA1_c_). In the 3rd quintile, correlation of HbA1_c_, triglycerides, and HDL-cholesterol occurred in the same direction (simultaneous increase in HbA1_c_, triglycerides, and HDL-cholesterol). In the 4th quintile, triglycerides increased alongside HbA1_c_. 

## Discussion

This population-based study showed distinct behavior of each class of serum lipids according to HbA1_c_ levels in individuals with type 1 diabetes. The risk of having low HDL-cholesterol did not show a homogeneous inverse relation to HbA1_c_, being lower in the middle range of HbA1_c_ (8.6 to 11.4%) than in HbA1_c_ levels equal or above 11.5%. The risk of high LDL-cholesterol and triglycerides levels both showed increase with worsening glycemic control, although in different thresholds of HbA1_c_. LDL-cholesterol worsened starting at 9.7% and triglycerides only above an 11.5% HbA1_c_ level. Regarding association with other variables, HDL-cholesterol showed marked inverse association with insulin dose. Rather than associating around a constant lipid factor, lipid variables correlated to glycemic control diversely according to HbA1_c_ level.

Not all previous studies have shown homogeneous behavior of HDL-cholesterol in type 1 diabetes. Some have reported worsening of HDL-cholesterol with poorer glycemic control [4,29], whereas others have demonstrated better HDL-cholesterol levels in type 1 diabetes when compared to non-diabetic controls [10,30]. Somehow in comparisons performed only among individuals with type 1 diabetes, patients with poorer glycemic control have also been shown to have a paradoxical elevation of HDL-cholesterol compared to well-controlled individuals [11,31]. Different stratification of HbA1_c_ levels may account for differences between our findings and existing literature. Some studies have stratified subgroups in HbA1_c_ levels as low as 7.5% [11], therefore being unable to assess differences in lipid behavior in the heterogeneous group of individuals with HbA1_c_ levels above 7.5. While a lower threshold of HbA1_c_ around 8% for changing behavior of HDL-cholesterol may be inferred from previous studies, an upper threshold is unclear. This also can account for the finding of worsening HDL-cholesterol with poor glycemic control. The grouping of individuals with intermediate HbA1_c_ levels, who would supposedly have higher HDL, with individuals with higher HbA1_c_, who would have lower HDL-cholesterol values, could result in average worsening of HDL-cholesterol without accounting for the heterogeneity in the whole spectrum of glycemic control. This differentiation is important since there is evidence that these higher HDL-cholesterol levels can be associated with higher cardiovascular risk in individuals with type 1 diabetes, lacking the protective effect of high HDL-cholesterol in non-diabetic individuals [6]. Another important aspect of low HDL-cholesterol is its relationship to higher insulin daily doses in type 1 diabetes therapy, which may indicate a higher insulin resistance background. Previous studies have shown IR to be a CVRF in individuals with type 1 diabetes [5]. Low HDL-cholesterol is traditionally associated to IR and hyperinsulinemia in non-diabetic individuals, albeit the causal relationship among these three alterations is unclear. Our findings show association of higher insulin dose with low HDL-cholesterol consistently, independently of glycemic control. In type 1 diabetes, IR is not an established etiological factor but it can progressively develop after clinical diagnosis [32]. Moreover, current insulin therapy methods are themselves a potential cause of hyperinsulinemia in these patients. Although the cross-sectional design of our study precludes any assumption of causality, one could postulate there is direct relationship between daily insulin doses and low HDL, without going through IR. The association of low-HDL-cholesterol with total daily insulin dose, corrected for BMI in the multivariable models (both regression and FA), could point to a role of IR by augmenting insulin needs, thus isolating the roles of obesity and exogenous insulin on HDL, rather than merging them both by using insulin dose per body weight instead. In this regard, a recent article has shown the insulin concentration required for 50% suppression of hepatic glucose production in a hyperinsulinemic/euglycemic clamp to be almost two times higher in type 1 diabetes than in controls adjusted for age, gender, and HbA1_c_. The authors suggest that hepatic and skeletal muscle IR in type 1 diabetes is not explained only by previously known factors [32]. Previous studies have already suggested the CV risk conferred by IR to be detached from lipid variables [5].

The finding of higher triglyceride levels with worse glycemic control appears to depend also on the mode patients are stratified for glycemic control. Studies that have divided patients in two groups with low HbA1_c_ thresholds have both showed no difference [10] or higher triglycerides in the higher HbA1_c_ group [11,29,31]. Again, the stratification of HbA1_c_ in lower levels can group together individuals with normal and high triglycerides, without necessarily establishing a threshold. Our data show a HbA1_c_ threshold for increase in the probability of hypertrigliceridemia above 11%. This value has not been clearly established by previous studies and possibly varies according to diet and population differences.

The inverse relationship of triglycerides and HbA1_c_ in normal controls and individuals with type 1 diabetes observed with HbA1_c_ below 7.5 is less well explained although it has been previously demonstrated [11,30,33]. Previous studies hypothesized that more intensive insulin therapy, which is usually the case in well-controlled patients, can bring lipids to values below those shown by normal controls. Insulin has effects upon lipid metabolism by stimulating enzymes such as hormone-sensitive lipase [34]. It is possible that in the normal (non-diabetic controls) or near-normal (good glycemic control) glucose range the effects of hyperinsulinemia can be more effective in controlling glucose as a compensatory mechanism than normal insulin values, thus mild degrees of hyperglycemia being associated with better triglyceride metabolism. A role for portal insulinopenia, as opposed to systemic hyperinsulinemia, cannot be excluded as well [35]. Nevertheless, the correlation of triglycerides with the Hyperinsulinemia/IR factor hypothesized in our FA still leaves the order in the causal relationship of IR and hyperinsulinemia open to questioning.

LDL-cholesterol metabolism showed a pattern similar to triglycerides, although with a lower threshold. This finding furthermore supports the view that lipids are influenced by glycemic control of type 1 diabetes in a complex manner, not interacting with other cardiovascular risk factors such as blood pressure and obesity analogously to the CVRF cluster of type 2 diabetes.

Clustering of CVRF has been analyzed by factor analysis in various contexts. Its reproducibility has been criticized, since many reported models have yielded different results. Since this statistical technique (or any other, for that matter) is unable to assess biological plausibility of the models, one has to take especial care on previous planning of the analyses rather than in interpreting them. One-factor models of CVRF clustering are pathophysiologically implausible, given the multifactorial nature of involved conditions. The heterogeneity of previous results, therefore, can be attributed to differences in the populations analyzed and in the variables entered in the FAs. Glycemic control has not been frequently assessed previously in the clustering of CVRF in type 1 diabetes [23]. We have previously demonstrated correlation of lipids and HbA1_c_ in type 1 diabetes by means of factor analysis, but without the necessary statistical power to divide the 520 patients in subgroups according to HbA1_c_ levels [24]. From this point of view, the present study is adequately powered to perform the analyses, once the subgroups have samples above 200 hundred individuals, considered adequate by most authors [36]. Regarding reproducibility, FA is an adequate method to test our hypothesis of different clustering of CVRF according to HbA1_c_ level, since individuals form the same population have been compared using exactly the same variables.

The most important limitation of our study is its cross-sectional design, unable to assess temporal relationship among the various factors studied. Another limitation is that FA was not performed separately by gender. Gender significantly influenced the frequency of low-HDL-cholesterol, high-LDL-cholesterol, and hypertriglyceridemia in the logistic regression models, but dividing the five quintiles of HbA1_c_ in ten groups by gender would impair sample power for performing FA. Another alternative approach would be using wider intervals of HbA1_c_ to avoid excessive number of subgroups. In our view, this approach would generate more heterogeneous groups regarding glycemic control and would be inadequate to test our hypothesis. Nevertheless, gender differences in CV risk seem to be attenuated or even erased in type 1 diabetes [5], making our FA model without subdividing by gender valid to assess the main hypothesis of this paper. The absence of direct measurements of IR is also an important limitation, although it wouldn’t necessarily be feasible in such a large sample.

## Conclusions

We present data from a large multi-centre cross-section study of type 1 diabetes in the Brazilian population, providing new insight on how glycemic control may influence behavior of dyslipidemia in type 1 diabetes, individually for each lipid fraction. Different levels of HbA1_c_ are significantly associated with change in fasting lipids, but a threshold of HbA1c beyond which lipid variables start to change is not homogenous, challenging the view of CVRF clustering as a single pathophysiological construct in type 1 diabetes. These considerations are important to decide when medical therapy may be required to optimize lipid and cardiovascular health besides diet, lifestyle interventions, and glycemic control level in individuals with type 1 diabetes, sometimes already at an early age.

Further prospective studies assessing cardiovascular events and intervention studies to evaluate if glycemic control also influences response to medical treatment of dyslipidemia are warranted to scrutinize the impact of the present information on actual cardiovascular prognosis of patients with type 1 diabetes.

## Abbreviations

CVRF: Cardiovascular risk factors; FA: Factor analysis; IR: Insulin resistance; MBP: Mean blood pressure; NCEP-ATP III: National Cholesterol Education Program-Adult Treatment Panel III.

## Competing interests

All authors declare that they have no competing interests.

## Authors’ contributions

FMAG conceived the study, performed the statistical analyses and drafted the manuscript; ADG, PD, OSM, RMCF, RC, CAN, and MBG collected data on individuals with type 1 diabetes and provided critical appraisal during the drafting of the manuscript; ERR and DBM collected data on non-diabetic individuals and provided critical appraisal during the drafting of the manuscript; SAD conceived the study, drafted the manuscript, collected data on individuals with type 1 diabetes, supervised the project, and is the guarantor of this study. All authors read and approved the final manuscript.
